# Supplementary material for: Pharmacokinetic and Pharmacodynamic Analysis of the Oxacephem Antibiotic Flomoxef against Extended-Spectrum β-Lactamase-Producing Enterobacterales from Dogs
Source: Int J Mol Sci. 2024 Jan 16;25(2):1105. doi: 10.3390/ijms25021105 (PMC10816067; doi:10.3390/ijms25021105)
Supplement: Supplementary file 1 [file ijms-25-01105-s001.zip › ijms-2810646-supplementary.pdf]

Table S1. Summary of PTA (%) and nonclinical PK-PD cutoff values of FMX at 30, 40, and 50 mg/kg in dogs.

Dose	Dosing interval	Minimum inhibitory concentration (μg/mL)[1]												Nonclinical PK–PD cutoff values
		0.031	0.0625	0.125	0.25	0.5	1	2	4	8	16	32	64	
30 mg/kg	q12h	**99.47**	**98.92**	**97.90**	**94.65**	86.09	65.74	32.05	4.91	0.10	0.00	0.00	0.00	≤ 0.25
	q8h	**99.98**	**99.91**	**99.90**	**99.76**	**99.34**	**97.79**	**92.65**	73.44	27.70	1.28	0.00	0.00	≤ 2
	q6h	**100.00**	**100.00**	**100.00**	**99.99**	**99.94**	**99.79**	**99.42**	**96.48**	79.72	24.93	0.56	0.00	≤ 4
40 mg/kg	q12h	**99.79**	**99.36**	**98.54**	**96.25**	**90.93**	75.51	46.52	13.04	0.75	0.00	0.00	0.00	≤ 0.5
	q8h	**99.98**	**99.95**	**99.86**	**99.83**	**99.62**	**98.71**	**95.69**	84.34	49.17	6.68	0.02	0.00	≤ 2
	q6h	**100.00**	**100.00**	**100.00**	**100.00**	**99.95**	**99.89**	**99.55**	**98.32**	**90.38**	50.85	4.09	0.04	≤ 8
50 mg/kg	q12h	**99.79**	**99.47**	**98.81**	**97.32**	**93.24**	81.89	57.73	22.94	2.42	0.02	0.00	0.00	≤ 0.5
	q8h	**99.99**	**99.98**	**99.93**	**99.86**	**99.77**	**99.12**	**97.56**	89.55	63.75	16.97	0.36	0.00	≤ 2
	q6h	**100.00**	**100.00**	**100.00**	**100.00**	**99.96**	**99.92**	**99.76**	**99.04**	**94.48**	69.54	11.45	0.10	≤ 8

[1] All of the PTA values of ≥ 90% were described in boldface. Only PTA values at nonclinical PK-PD cutoff were surrounded by frame lines.

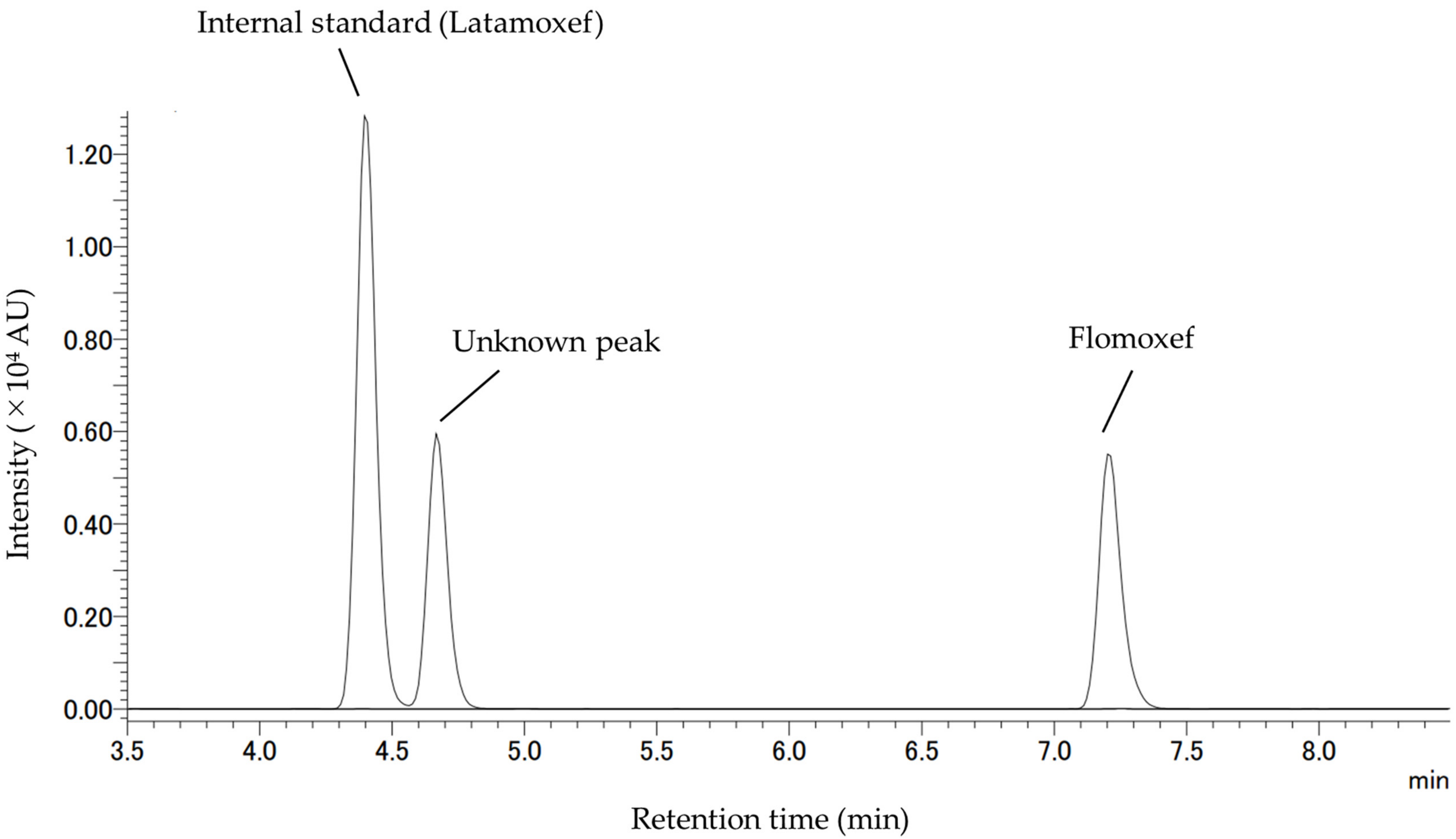

Figure S1. Representative chromatogram of flomoxef (1 μg/mL) and internal standard (latamoxef) in the LC-MS/MS method established in this study.
